# A promising tool for surgical lipotransfer: a constant pressure and quantity injection device in facial fat grafting

**DOI:** 10.1186/s41038-017-0077-9

**Published:** 2017-05-31

**Authors:** Mei Song, Yi Liu, Ping Liu, Xianying Zhang

**Affiliations:** Burns and Plastic Surgery Center, General Hospital of Lanzhou Military Command of the PLA, 333 South Riverside Road, Lanzhou, 730050 Gansu China

**Keywords:** Facial fat grafting, Fat granule, Invasive

## Abstract

**Background:**

The purpose of this study was to preliminarily assess a constant pressure and quantity fat granule injection device for minimal invasive properties in clinic.

**Methods:**

A retrospective controlled study was carried out, from October 2013 to January 2015, on 76 female healthy patients aged between 26 and 53 years at the General Hospital of Lanzhou Military Command, China. To achieve small volume, high thrust, and precision requirements of facial fat grafting, an integrated handheld controller and motor design was adopted, reducing the volume and weight of the fat transplantation injection device. The 76 patients underwent 90 procedures each side of the face; each patient was treated with the aforementioned device on the face’s left side, while a conventional hand-push injection device was used on the right side as control. The outcome was assessed on pre- and postoperative images, with 6-24 months follow-up.

**Results:**

The current device consistently allowed deposition of fat threads at about 55 μL/cm after cannula withdrawal; the volume of fat injected could be precisely adjusted to 0.04 mL/s. This device had the advantages of small-volume injection and convenient operation. The patients exhibited a good, stable shape and a smooth contour line in both sides. The long term satisfaction was higher for the left side than for the right one. Nodules and unevenness occurred only on the right side. Ecchymosis occurred significantly less frequent on the left side than the right one. Intraoperative pain was significantly lower for the left side than the right one.

**Conclusions:**

This device offered superior control compared with the conventional one and constitutes a promising tool for surgeons practicing lipotransfer.

**Electronic supplementary material:**

The online version of this article (doi:10.1186/s41038-017-0077-9) contains supplementary material, which is available to authorized users.

## Background

Autologous fat grafting has helped for more than a century treat various conditions, such as syndromic defects, posttraumatic and postoperative complications, velopharyngeal insufficiency, and esthetic disorders, with the technique gaining increasing popularity in the last 25 years [1]. However, no consensual approach exists as for the best technique to be used. Various aspects of fat grafting have been studied, including covering the donor site, aspiration methods, local anesthesia, fatty tissue processing, reinjection, and graft stability. Unfortunately, many proposed techniques were not validated in well-designed clinical trials. Meanwhile, the final outcomes evaluated usually lack quantitation of fat grafting viability [1, 2].

Carpaneda et al. [3] found 40% graft survival at 1.5 ± 0.5 mm around the graft margin, with thickness and shape determining fat transplantation success; they proposed to use fat grafts with diameters < 3 mm for high graft survival [4]. Coleman [5] conceived the notion of structure fat grafting, insisting fat parcels ought to be manually placed with < 1/10 mL injection volume per parcel.

To enhance grafting survivability, small parcels of fat must be distributed evenly throughout the recipient tissue; the parcels should be small enough for plasmatic imbibition to occur, thereby providing sufficient nutrition to support the survival of fat grafts until neovascularization develops. Therefore, injection equipment and techniques are particularly important in optimizing the clinical effects [6].

To consistently inject a fat thread at every cannula passage, a constant pressure and quantity injection device was developed. The device releases a set volume of filler material per unit distance after cannula withdrawal. The current study aimed to describe the architectonic of the injection device, which was also assessed as an automated tool in facial fat grafting.

## Methods

### Device description

**Fig. 1:**
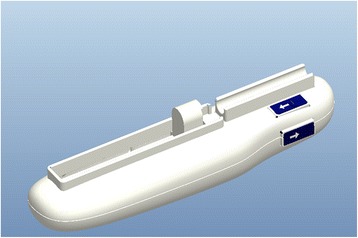
Injection device (Reprinted with the permission from Zhonghua Zheng Xing Wai Ke Za Zhi) [7]

**Fig. 2:**
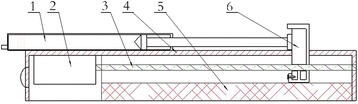
Internal structure of the injection device (*1* syringe, *2* micro DC motor, *3* lead screw, *4* shell, *5* battery, *6* injection handle) (Reprinted with the permission from Zhonghua Zheng Xing Wai Ke Za Zhi [7])

The device is a hand-held electric precision injection system (YSZTQ-01, Lanzhou Wenhe Medical Instrument R&D Co., Ltd, Lanzhou), with a motor as the power source driving the slider movement through screw rotation to impel the injection syringe’s plunger (Fig. 1) (Reprinted with the permission from Zhonghua Zheng Xing Wai Ke Za Zhi) [7]. It uses a standard, commercially available 1-mL disposable syringe (WEGO, Weihai, China). The syringe carrier or device body holds the cylinder securely. A micro DC motor controls the front part of the circuit (Fig. 2) (Reprinted with the permission from Zhonghua Zheng Xing Wai Ke Za Zhi) [7]; it incorporates a control circuit, which controls the motor speed and screw rotation, causing fat to be injected at different rates. The micro DC motor is connected through coupling with a screw end in the rear part. The other screw end is connected with the sliding block. The syringe is clipped into the device’s buckle; the control circuit monitors the current to the motor, enabling it to drive screw rotation, while the sliding block moves at a constant speed, pushing the plunger forward for injection. The syringe carrier includes a velocity/reset button, which can cause automatic slide block retraction, remaining in such position until syringe lipping; the device is therefore ready for another injection.

The front part of the device consists of a bionic design rack, which allows a firm grip by the operator. The rear portion has a 3.6 V rechargeable lithium battery, which can be used repeatedly. The device is made of stainless steel and acrylic materials, is reusable, and uses cobalt 60 as a source for radiation disinfection or low temperature plasma sterilization.

The device injects 55 μL of filler material, and cannula withdrawal from tissue was performed every 1 cm. The amounts of fat injected can be accurately adjusted to 0.04 mL/s, somewhat below the internal volume of a 12-gauge cannula. Consistent fat threads were produced in the recipient bed thanks to uniform distribution.

### Patients

A retrospective controlled study was carried out from October 2013 to January 2015 on 76 healthy female patients aged between 26 and 53 years at the General Hospital of Lanzhou Military Command, China. Approval was obtained for the current study from the hospital’s Ethics Committee. All patients who underwent facial fat grafting provided informed consent allowing the device described below to be used.

The device was not used in the following situations: chemotherapy, radiotherapy, prolonged steroid use, connective tissue or chronic blood ailments, and systemic metabolic disorders. The patients were also excluded with asymmetric facial features.

### Fat grafting

A tumescent technique with a 10-mL syringe under low-pressure suction harvest was employed. The abdominal area was most commonly employed as the donor site. Other sites included thighs or buttocks. The syringe was kept upright until fat separation to yield a distinct yellow layer, which was collected. The wicking method was employed to remove oil and nonviable fat. The collected fat was then sampled into 1 mL syringes for injection.

**Fig. 3:**
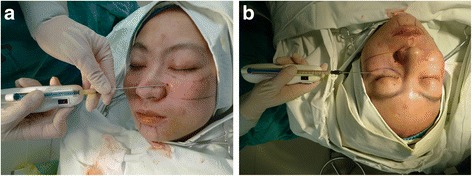
The device injecting fat into the (**a**) nasojugal, (**b**) palpebromalar



In this study, a total of 90 facial fat-grafting procedures were performed on each side of the face of the 76 subjects, with 6 month of follow-up or more. Thirty-eight patients underwent procedures for correcting grooves and creases, including 24 nasolabial fold, 6 glabella, and 8 nasojugal groove cases. Fifty-two patients underwent fat transfer for volume augmentation: 31 malar, 7 lip, 8 temple, and 6 chin cases. Each patient was treated using the aforementioned device on the face’s left side, with conventional hand-push injection used on the right side as a control. The device operates by first stabilizing the tissue and by advancing the cannula into it, and then depositing the fat by pressing the velocity button as the cannula is withdrawn. The cannula is allowed to stay in the same place for a longer duration to deposit more fat in the case of large treatment areas (Fig. 3). (See Additional file 1: Video S1, which displays the autologous fat transplantation for frontal. This video is available in the “Additional file 1” section of the full-text article). Structural fat grafting [8] was employed, injecting equivalent amounts of fat on both sides of the face.

**Additional file 1:** The autologous fat transplantation for frontal. (MP4 1618 kb)


### Data

Images were acquired before operation, right after surgery, and at 6-24 postoperative months. Position, facial expression, focal distance, and camera configurations were all standardized. Patients, plastic surgeons, and a third party unrelated to the study were required to evaluate the therapeutic effects, which included: (1) general level of satisfaction for each side; (2) notable asymmetry reflected by treated area volumes; (3) nodules, unevenness, ecchymosis, and pain; (4) skin texture amelioration.

Chi-square test and Fisher's exact test were employed to statistically assess the aforementioned points. Significance level was set at *P* < 0.05.

## Results

**Fig. 4:**
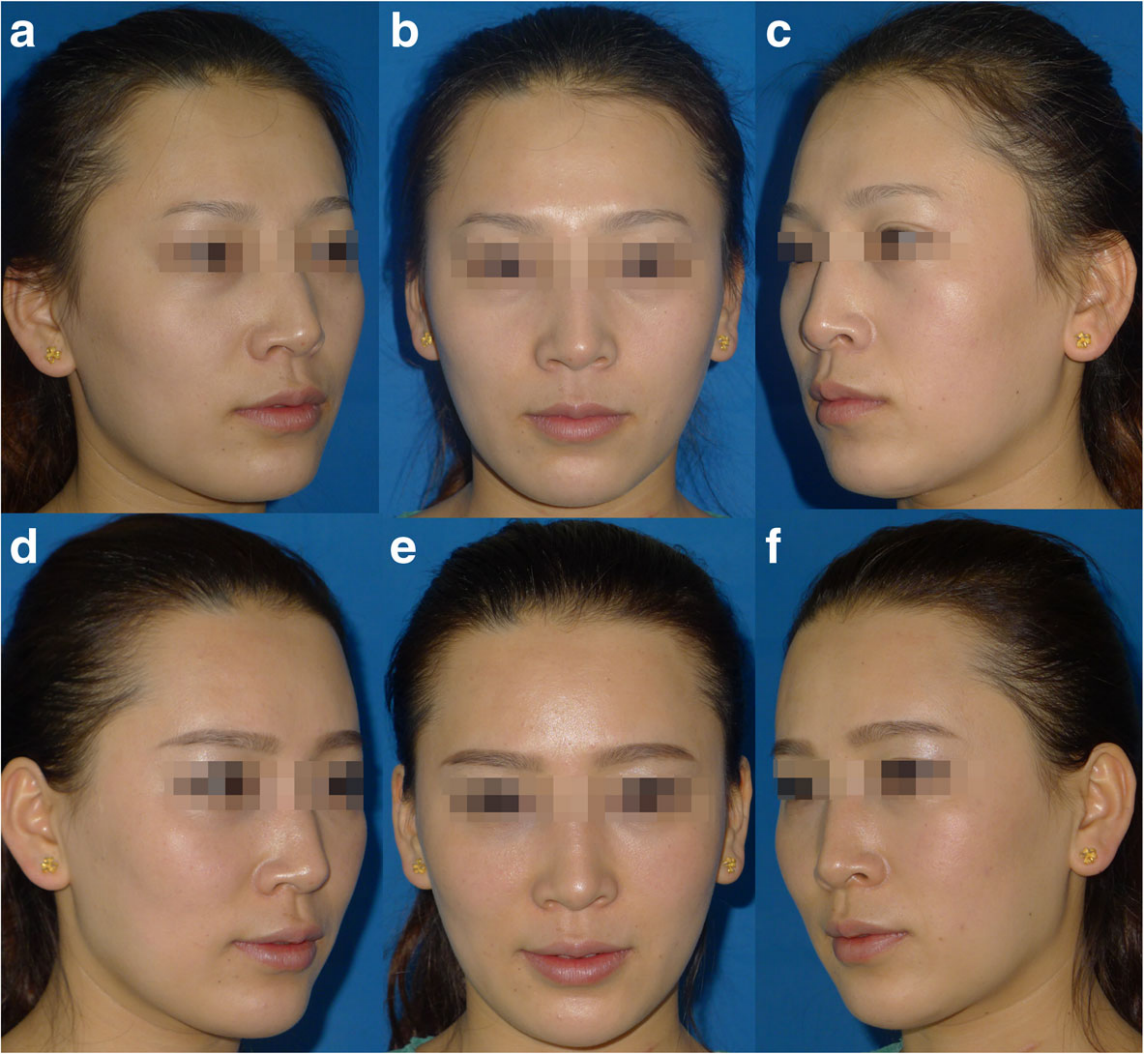
A 32-year-old patient receiving fat grafting with the device (*left face*) and the traditional hand-push injection (*right face*): (**a**–**c**) Preoperative view. (**d**–**f**) Postoperative view 10 months after fat grafting to the frontalis, tear trough areas, submalar region, nasolabial and marionette folds, lower mandible, and lips. The contour line of the left cheek and mandible is smoother and softer than the right side

**Fig. 5:**
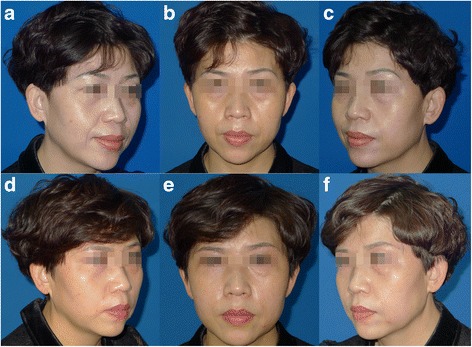
A 45-year-old patient receiving fat grafting with the device (*left face*) and the traditional hand-push injection (*right face*): (**a**–**c**) Preoperative view. (**d**–**f**) Postoperative view 14 months after fat grafting to the submalar region and nasolabial folds. The *left cheek* and nasolabial folds still plump

Plain photographs (Figs. 4 and 5) were solely used to assess esthetic results, without the aid of any imaging diagnosis (ultrasound, magnetic resonance imaging, or three-dimensional scanning).

The 76 patients were followed-up for 6-24 months. All patients with grafting of one to three facial regions received fat injections once or twice, with an injection volume for each region of 0.05–26.43 mL. The average follow-up period after the final injection was 10.7 months. During the observation period, the patients exhibited a good, stable shape and a smooth contour line. Three patients reported a low level of satisfaction bilaterally, and complained of fast absorption. Furthermore, only two patients reported to have noted a volume difference on the two sides. The overall satisfaction was higher for the left side than for the right one (Table 1). Nodules and unevenness occurred only on the right side. Ecchymosis occurred bilaterally, but was significantly less frequent on the left side than the right one (Table 1). Intraoperative pain was significantly lower for the left side than the right one (Table 1). No complications were recorded at either donor or recipient sites.

**Table 1:** Comparison of two groups of patients for treatment evaluation *n* (%)

Evaluation content	Options	Left side (using the device) n(%)	Right side (using the conventional technique) n(%)	*P* value
1. General level of satisfaction	Good	76 (84.4)	58 (64.4)	0.005
	Sufficient	11 (12.2)	29 (32.2)	
	Bad	3 (3.3)	3 (3.3)	
2. Notable asymmetries in terms of volume	Yes	1 (1.1)	1 (1.1)	1.000
	No	89 (98.9)	89 (98.9)	
3. Notable irregularities	Nodules	0 (0)	6 (6.7)	0.029
	Unevenness	0 (0)	11 (12.2)	0.001
	Ecchymosis	28 (31.1)	42 (46.7)	0.032
	Thickened areas	6 (6.7)	8 (8.9)	0.782
	Pain	38 (42.2)	62 (68.9)	0.001
4. Level of improvement in skin texture	High	72 (80.0)	65 (72.2)	0.221

## Discussion

Autologous fat grafting is commonly used for correcting anomalies (contours) in birth defects, trauma sequelae, surgery rejuvenation, and burn injuries. Burn sequelae result in local volume loss and skin scarring. Autologous fat grafting can restore a missing relief by filling a localized depression, reshaping the face volume, or smoothing a scarring skin [9, 10]. Although autologous fat transfer usually generates optimism in clinic, there is uncertainty concerning the graft viability [11].

In the past decades, fat-grafting procedures have undergone important changes to improve fat survival or retention. Gonzalez et al. [12] advocated the gap formed by a puncture prior to injection; Barret et al. [13] and Coleman [5] proposed multichannel, multipoint, and small amount injections using a 1-mL or 5-mL syringe or thin sleeve; Xie et al. [14] developed the “3L3M” autologous fat transplantation technique. Adopting a multilayer, multiway tunnel that can improve the survival rate has become the consensual view. Injecting the same small volume of fat is very important. Too much would kill fat cells and would cause inflammation and edema with inconsistent outcomes. The conventional hand-push injection method hardly controls the injection amounts and prolongs postoperative edema. In addition, it could cause needle- and cannula-associated problems of clogging. This leads to the surgeon increasing the syringe pressure, which not only enhances the fat amounts administered to unintended areas, but also can cause damage to fat cells [6]. Moreover, it is difficult to administer a consistent thread of fat after cannula withdrawal; therefore, fat deposits tend to form clumps, with irregular distribution and subsequent necrosis. Because of fine anatomic and complex morphological structure, facial appearance requirements are higher. Considering the stereo frame structures of the dermis, ligaments, and muscles, a scientific comparison of the fat fine grafting technique and grafting tools is needed.

To inject consistent threads of fat at every cannula passage, multiple innovative techniques have been proposed for ameliorating fat grafting [15–20]. Fulton et al. [21] attached the syringe to the scale of the fat grafting gun and pulled the trigger once the syringe pushed forward a grid (of 0.1 ml); although accurate, the injection quantity was too high. Yoshimura et al. [22] separated the syringe from the needle, had the syringe pushed by a specialized doctor while other physicians controlled the needle’s movement, so as to achieve constant force and pressure injection. Hetherington et al. [23] invented a prototype injection control device driven by a motor using a series of transmission gears to precisely control the speed and quantity of injection. In recent years, the commercially available micro-autologous fat transplantation (MAFT) device invented by Lin et al. [24] has been employed for fat grafting. MAFT’s advantage is to convey fat parcels of accurately controlled volumes per injection. However, this invention was based on the burst mode in which the operator needs to constantly pull the trigger, resulting in fatigue in fingers, human consumption, and bolus incoherence. Moreover, in some burn sequelae, tight local fibrous adhesions will increase the injection pressure; the burst mode is affected by the interference of pressure, and consistent fat parcel deposition cannot be achieved.

To avoid these shortcomings, the current study independently developed a constant pressure, constant quantity, and minimally invasive fat transplantation injection device. In the newly engineered device, an electric drive was adopted through a gear motor drive reducer, with a screw rotation drive pushing the syringe handle forward at a constant speed. The electric drive is not subject to pressure interference during injection, as it can continue to maintain a constant speed. A current limiting device was included in the circuit board with self-protection effects, to prevent injecting the bolus too fast, so as to maintain a uniform bolus. This injection device can dispense extremely small and uniform threads of fat with high precision, achieving constant injection and minimizing tissue trauma. With 1-mL syringes, it injects 56 μL of fat granules every centimeter after cannula withdrawal. Using this tool, uniform thread-shaped fat is always delivered into the recipient bed. Also, variable tissue resistance would not affect fat injection since the device is equipped with an electric drive.

The proposed device prevents tissue trauma. With equal fat amounts administered, the volume of fat in every unit length tunnel is small; this results in low local tissue tension, therefore, decreasing pain sensation. This technique has shifted the focus of fat injection from operation skills to accurate and quantified fat delivery, thereby making fat grafting easy, efficient, and timesaving.

In this study, the minimum injection site area was about 1.0 cm^2^, with a minimum filling volume of only 0.05 mL. It is difficult for a conventional hand-push injection, which relies solely on the surgeon’s experience. to control the filling amounts and to inject so limited fine fat filling. This device can be used to precisely control the filling volume, shorten operative time, and reduce tissue damage and bleeding, thereby, significantly reducing pain and reducing the surgeon’s efforts.

Here, one half of the face was treated with the developed device, while the other underwent a conventional hand-push injection. As shown above, the clinical effectiveness of the current device was superior to that of the syringe-push mode; nodules, unevenness, ecchymosis, and pain were significantly reduced, with higher satisfaction of patients at clinical follow-up. Because of these findings, the authors adopted this fat transplantation injection device after years of using the conventional hand-push injection.

## Conclusions

The constant pressure and quantity injection device proposed in this study yields higher overall satisfaction than the conventional hand-push injection tool. More consistent and clinical esthetic results, faster recovery, and decreased operation time were observed in comparison with conventional hand-push injection. One may therefore conclude that this device is important for lipotransfer.
